# Behavioral normalization and social media influence: perceptions and impacts among Moroccan users

**DOI:** 10.1192/j.eurpsy.2026.11493

**Published:** 2026-07-31

**Authors:** S. Essadki, F. Wahoud, W. Hajjaji, A. Zarouali, R. Lamsyah, S. Bechrouri, S. Jbara, M. Hamlili, S. Salhi, M. El Gardid, M. A. Lafraxo

**Affiliations:** 1 Higher Institute of Nursing Professions and HealthTechniques; 2 Laboratory of Maternal, Infant, and Mental Health(LSMIM), Faculty of Medicine and Pharmacy, Oujda; 3 Higher Institute of Nursing Professions and HealthTechniques, Fez; 4 Laboratory of Biology and Health, Faculty of Science, Ibn Tofail University, Kenitra, Morocco

## Abstract

**Introduction:**

In the context of the digital expansion and the growing influence of social media, influencers play a major role in shaping social norms and modifying the behaviors of their users.

**Objectives:**

This cross-sectional observational study aims to identify the behaviors encouraged by content creators among the Moroccan population, as well as the factors associated with trust levels and the perceived influence of consumption recommendations.

**Methods:**

An online questionnaire was distributed. A total of 1,006 active social media users aged 18 years and older from various cities across Morocco were recruited. The data were analyzed to identify positive and negative behaviors encouraged by influencers, as well as the factors determining trust and perceived influence.

**Results:**

Data analysis shows that more than half of the respondents believe that influencers contribute to the dissemination of social norms. Regarding negative behaviors, 59.6% of participants reported the normalization of provocative clothing, 39.6% indicated psychological pressure related to social comparison, and 34.6% acknowledged the promotion of a culture of superficiality. Conversely, positive behaviors cited include the popularization of scientific content (47.3%), awareness-raising of appropriate behaviors (40.5%), and the promotion of a healthy lifestyle (37.2%). Factors significantly associated with trust in influencers and perceived influence include content quality and the themes addressed, regardless of the domain (fashion, beauty, sports, nutrition, education, travel, personal development).

**Conclusions:**

This study highlights the tangible impact of influencers on behavioral and social practice changes among Moroccans. These findings underscore the importance of strengthening digital literacy and promoting the rational and responsible use of social media

**Disclosure of Interest:**

None Declared

